# Acquired Gastric Dieulafoy-Like Lesion due to Aberrant Blood Supply Diverted From the Left Phrenic Artery to an Enlarged Splenule

**DOI:** 10.14309/crj.0000000000001032

**Published:** 2023-04-14

**Authors:** Benjamin Lew, Dennis E. Der, Brian S. Lim

**Affiliations:** 1 Kaiser Fontana Medical Center, Fontana, CA; 2 Kaiser Riverside Medical Center, Riverside, CA; 3 Kaiser Los Angeles Medical Center, Los Angeles, CA; 4 University of California Riverside School of Medicine, Riverside, CA; 5 United Gastroenterologists, Irvine, CA

**Keywords:** Dieulafoy, gastric varices, esophagogastroduodenoscopy, splenectomy, embolization

## Abstract

Dieulafoy lesion is an aberrant submucosal vessel that can erode into the overlying tissue leading to hemorrhage. It is a rare but important cause of gastrointestinal bleeding. We present a case of a patient who developed an acquired Dieulafoy lesion 39 years after splenectomy. Abdominal computed tomography showed an aberrant vessel from a branch of the left phrenic artery, coursing through the gastric fundus to supply a splenule. Angiography with embolization of the aberrant vessel resulted in no further bleeding.

## INTRODUCTION

Dieulafoy lesion (DL) is a rare cause of gastrointestinal (GI) bleed, accounting for 1.5%–5% of cases. It was first discovered by Gallard and characterized by Georges Dieulafoy.^1,2^ DL is an aberrant artery that courses and erodes through the submucosa of the GI tract.^3^ Unlike normal submucosal arteries, DL maintains a large caliber throughout, with a typical diameter measuring up to 3–5 mm. Although it can occur anywhere along the GI tract, 75% are found in the stomach, most within 6 cm from the gastroesophageal junction along the lesser curvature.^4^

Although uncommon, DL is an important cause of acute GI bleed because it can lead to intermittent, brisk hemorrhage with 9%–13% mortality. DL can also present a diagnostic challenge given their small size and intermittent bleeding.^5^ DL hidden between folds, under adherent clots, or behind significant bleeding may be missed during endoscopy, requiring repeated endoscopies, cross-sectional imaging, and formal angiography for diagnosis.^6^

Majority of DLs are believed to be congenital, only causing clinically significant bleeding later in life after continued erosion through the submucosa and repetitive injury from pulsatile flow through the dilated artery.^3^ Acquired DLs are rare.

## CASE REPORT

A 52-year-old man presented with a 3-day history of melena with fatigue, diaphoresis, and lightheadedness. The patient's medical history was noncontributory except for splenectomy at age 13 from a car accident. He denied nonsteroidal anti-inflammatory drug usage, excessive alcohol consumption, tobacco, or illicit drug use.

In the emergency department, the patient was hemodynamically stable but demonstrated a decrease in hemoglobin from 17.7 to 10.3 g/dL in the prior 3 months. Esophagogastroduodenoscopy (EGD) revealed no esophageal varices but showed small, nonbleeding gastric varices (Sarin classification IGV-1). Colonoscopy was negative. Owing to patient's history of splenectomy and findings on EGD, abdominal computed tomography (CT) was performed. An enlarged, 8.3 cm splenule was seen as well as a diminutive splenic vein. CT also confirmed the presence of prominent perigastric vessels, but there were no signs of cirrhosis. Since the patient had no hematemesis and no blood in the stomach during EGD, capsule endoscopy was performed to rule out a small bowel (SB) etiology, which showed 3 small nonbleeding arteriovenous malformations (AVMs) in mid-SB along with brownish red residues suggestive of old blood. The patient's hemoglobin remained stable, and he was discharged with an outpatient referral for double-balloon enteroscopy (DBE). However, the patient did not keep his appointment for DBE because his symptoms resolved and was lost to follow-up.

**Figure 1.**
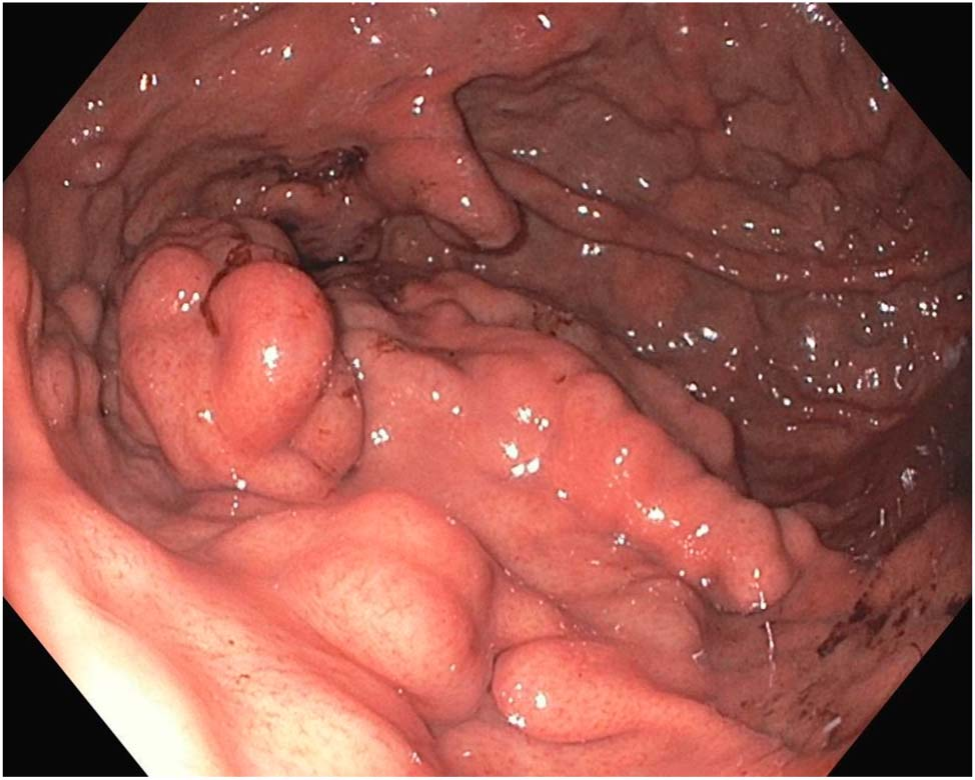
Esophagogastroduodenoscopy image of prominent vessels in gastric fundus pre-embolization.

**Figure 2.**
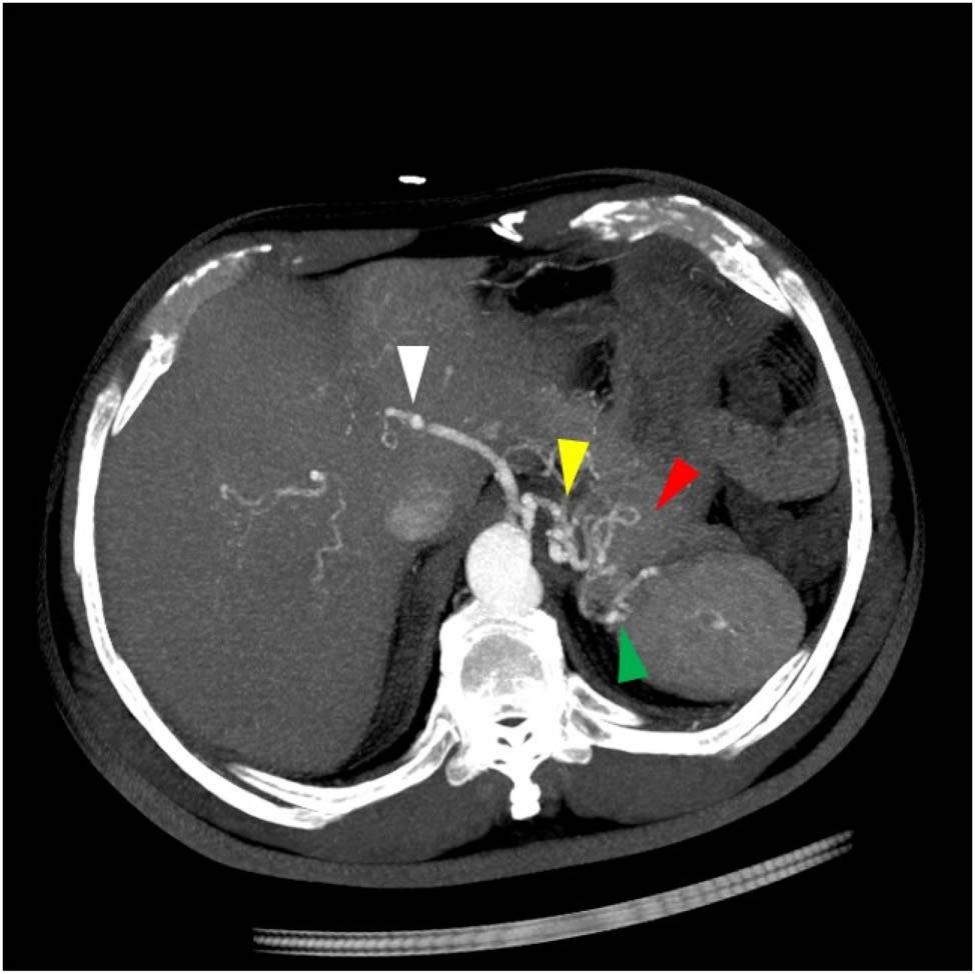
Oblique maximum intensity projection computed tomography image showing the course of the aberrant vessel, including the left inferior phrenic artery (yellow arrow) branch coursing into the stomach (red arrow) and to the splenule (green arrow). The celiac artery (white arrow) has no splenic branches.

**Figure 3.**
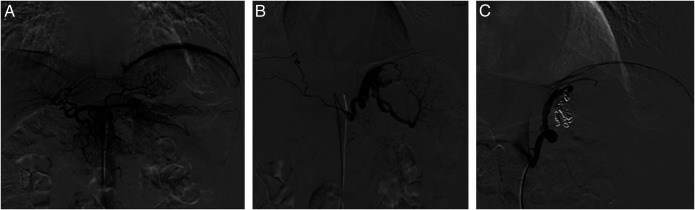
(A) Celiac angiogram showing no arterial supply to the splenule. (B) Inferior phrenic angiogram showing an enlarged, tortuous branch supplying the splenule. (C) Left inferior phrenic angiogram after coil and gel-foam embolization showing no residual flow through the aberrant artery.

**Figure 4.**
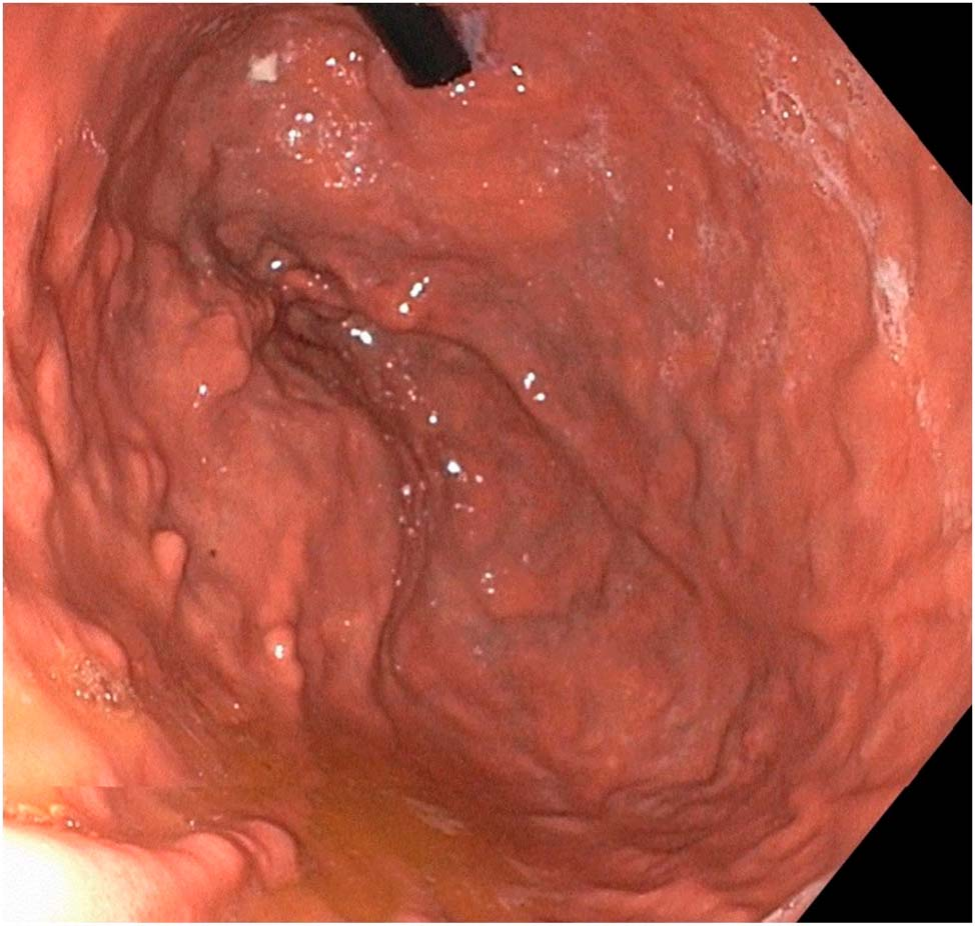
Repeat esophagogastroduodenoscopy postembolization demonstrating near resolution of prominent vessels.

The patient returned 9 months later with similar complaints. Repeat EGD revealed enlarged gastric varices, still without active bleeding or stigmata of recent bleed (Figure 1). Capsule endoscopy revealed fresh blood in the stomach tracking down into duodenum. Multiphase CT demonstrated an aberrant branch artery arising from the left inferior phrenic artery, traveling through the gastric fundus and supplying the large splenule (Figure 2). Based on this finding, the patient was referred to interventional radiology. Angiography was performed with coil and gel-foam embolization of the aberrant artery (Figure 3). It was concluded that SB AVMs seen on initial capsule endoscopy were incidental findings, and thus, DBE for treatment of these AVMs was deemed unnecessary. Repeat EGD 2 months after embolization demonstrated a significant decrease in number and size of the prominent fundic vessels which previously were believed to be “varices” (Figure 4). The patient is 4 years post embolization without recurrent episodes of GI bleeding.

## DISCUSSION

Dieulafoy lesion represents a large-caliber submucosal vessel that causes hemorrhage by either disrupting the overlying epithelium or becoming thrombosed and leading to tissue necrosis. Although the etiology of DL is poorly understood, most have been recognized as congenital. There have been few case reports of acquired etiologies such as Dieulafoy-like lesions of the bronchial arteries in the setting of chronic smoking^7^ and Dieulafoy-like AVM within a jejunal diverticulum.^8^ Although the association between DL and gastric malignancy is not fully elucidated, one theory states that the circulation disturbances from DL vessels causing repeated mucosal erosions and ulcer lead to regeneration and dysplasia, which advances to carcinogenesis.^9^

EGD is the preferred approach of investigation to determine the etiology of upper GI bleed including bleed from gastric DL. Typical endoscopic appearance of DL is a pigmented protuberance from a vessel stump with minimal surrounding erosions and no ulcerations. Actively bleeding DL usually demonstrates oozing or spurting blood. Alternatively, there may be a fresh adherent clot. DL should be strongly considered when a lesion is located in the proximal stomach and/or has a small mucosal defect connected by a narrow attachment point to an adherent clot. It is important to distinguish DL from other arterial lesions that can appear similar endoscopically including hereditary hemorrhagic telangiectasia, vascular neoplasms, angiodysplasia, aneurysms, and pseudoaneurysms.^4,10^

We present a case an acquired gastric Dieulafoy-like lesion 39 years postsplenectomy due to spleen trauma. The patient had an enlarged splenule with a hypertrophied blood supply diverted from the left phrenic artery. Aberrant artery arising from this traveled through the gastric fundus causing bleeding. Sikov et al also described a patient with GI bleed with a history of splenosis from traumatic splenic rupture. In their case, however, the source of bleeding was splenic implants along the SB serosa extending through the entire thickness of the bowel wall.^11^

The preferred initial treatment of gastric DL is mechanical endoscopic methods such as hemoclips and band ligation.^12^ Over-the-scope clips may also have a role because it is effective in various causes of severe GI bleeding.^13^ When endoscopic management fails, interventional radiology in the form of gel-foam embolization or laparoscopic/open surgical intervention may be indicated.^13^

In conclusion, our case highlights a few important aspects of upper GI bleed and DL. Although the initial EGD finding was believed to be gastric varices based on endoscopic appearance, an astute work-up revealed the final diagnosis of a rare arterial Dieulafoy-like lesion. In patients with a history of spleen trauma, it is important to consider acquired DL, especially in association with splenules. Finally, the case demonstrates the importance of multidisciplinary care in the accurate diagnosis and optimal management of GI bleed.

## DISCLOSURES

Author contributions: B. Lew wrote manuscript. DE Der contributed to writing manuscript and provided radiographic images. BS Lim oversaw manuscript, contributed to writing manuscript, provided endoscopic images, and is the article guarantor.

Financial disclosure: None to report.

Informed consent was obtained for this case report.
